# Supplementary figures and images for: Mesenchymal stromal cell chondrogenesis under ALK1/2/3-specific BMP inhibition: a revision of the prohypertrophic signalling network concept
Source: Stem Cell Res Ther. 2024 Apr 5;15:98. doi: 10.1186/s13287-024-03710-7 (PMC10998299; doi:10.1186/s13287-024-03710-7)

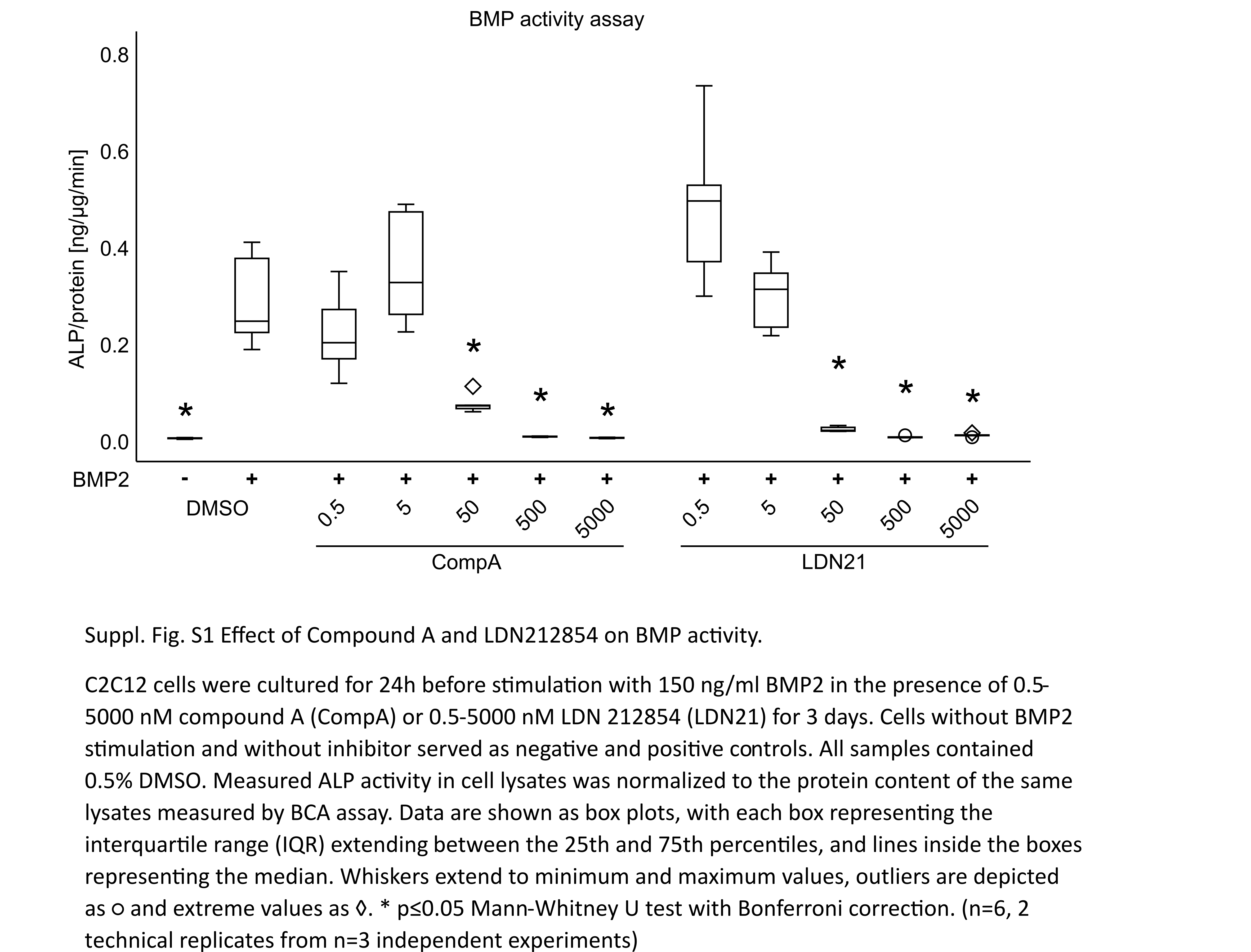

Supplement: Supplementary file 1 — Supplementary Material 1 [file 13287_2024_3710_MOESM1_ESM.png]

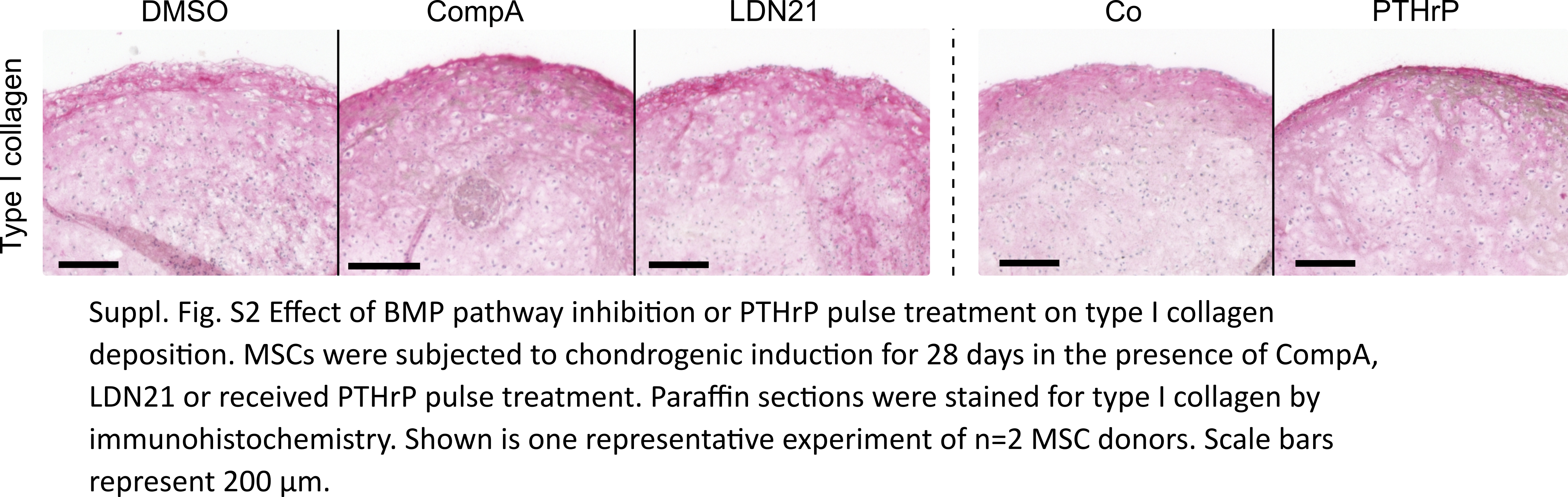

Supplement: Supplementary file 2 — Supplementary Material 2 [file 13287_2024_3710_MOESM2_ESM.png]

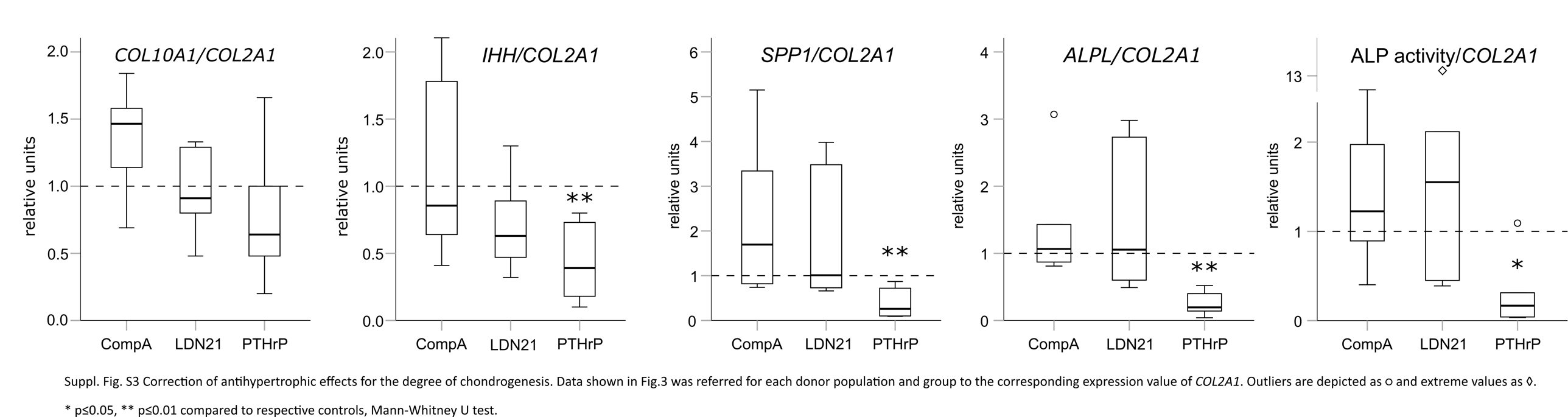

Supplement: Supplementary file 3 — Supplementary Material 3 [file 13287_2024_3710_MOESM3_ESM.png]

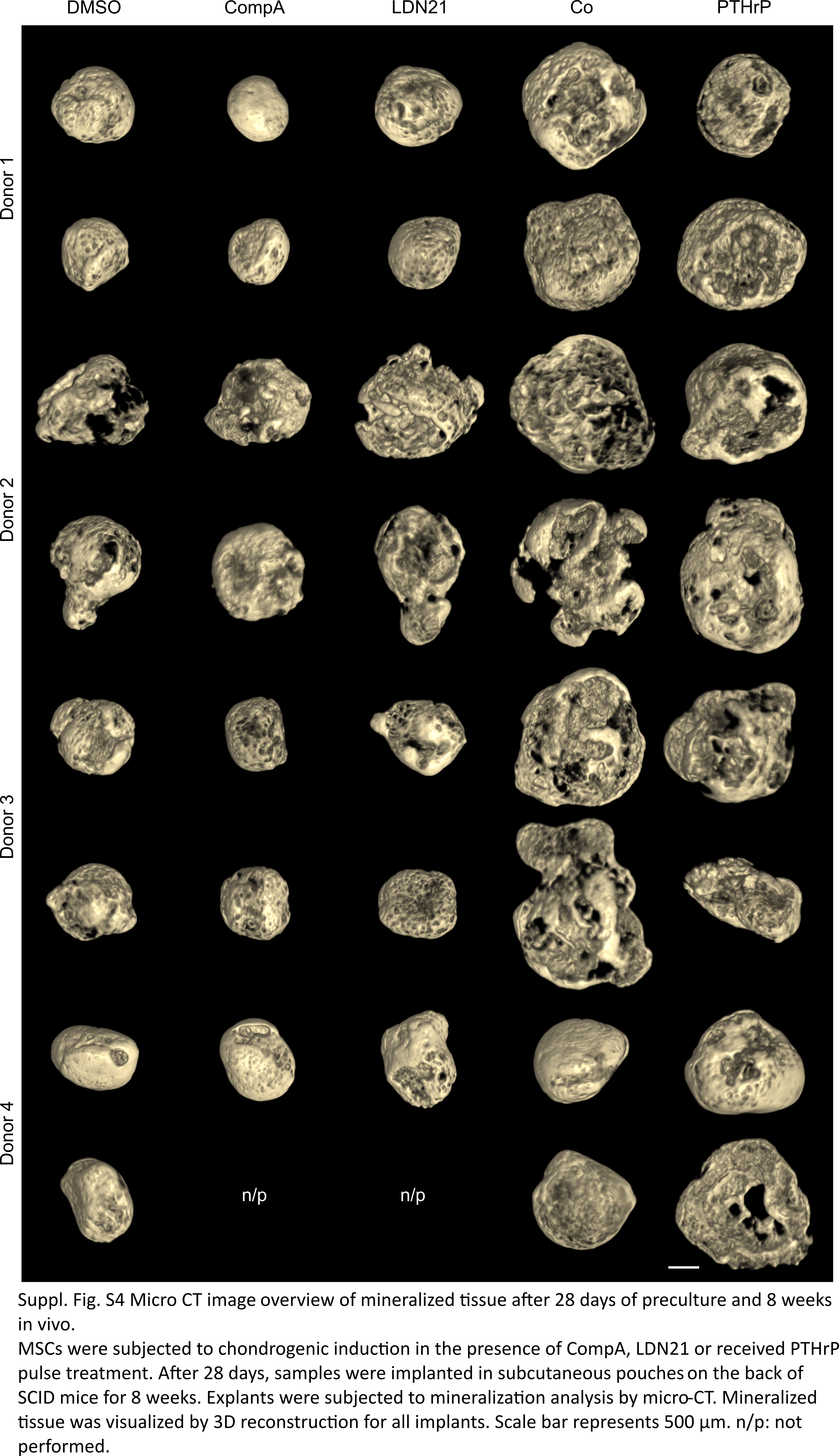

Supplement: Supplementary file 4 — Supplementary Material 4 [file 13287_2024_3710_MOESM4_ESM.png]

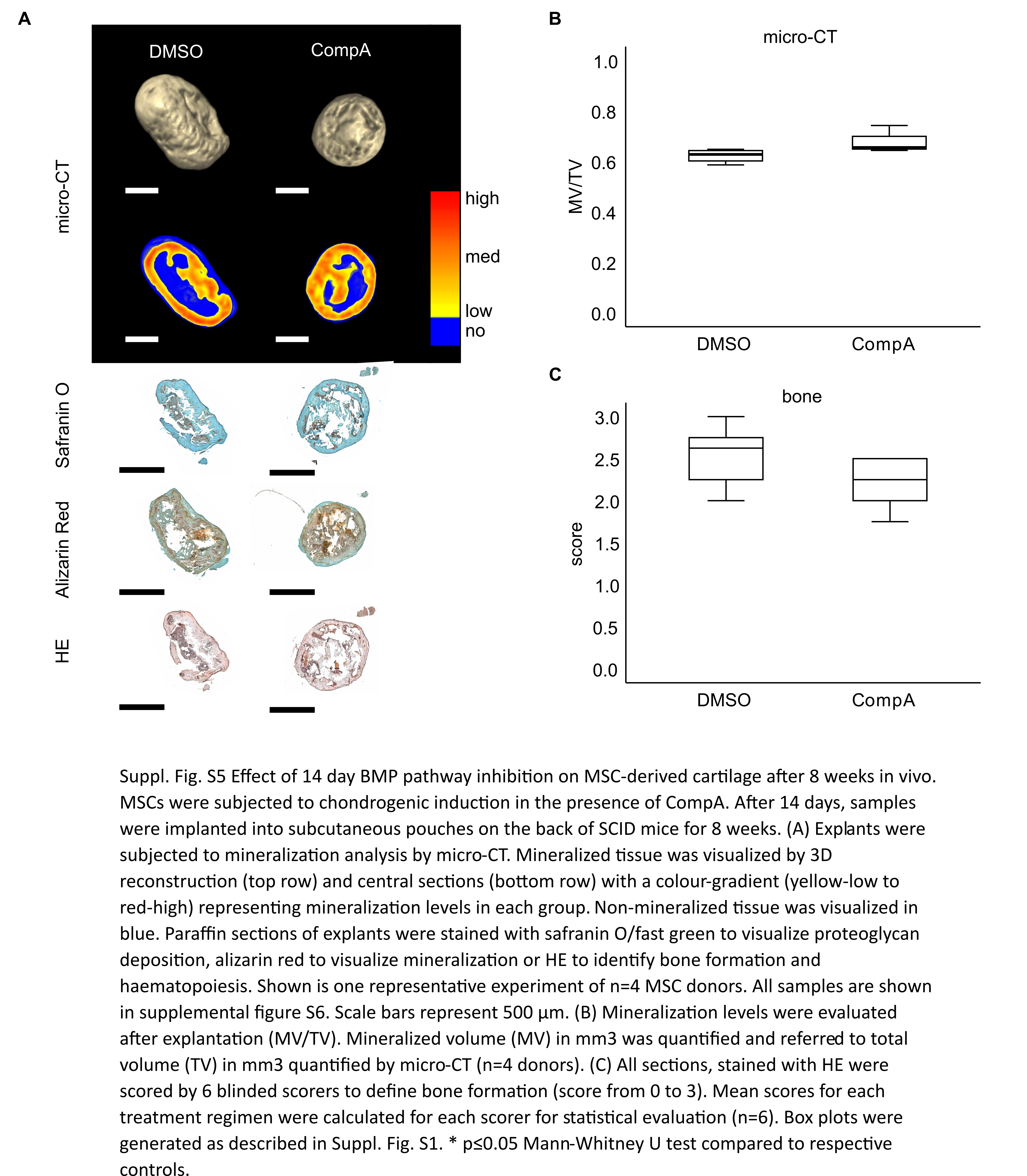

Supplement: Supplementary file 5 — Supplementary Material 5 [file 13287_2024_3710_MOESM5_ESM.png]

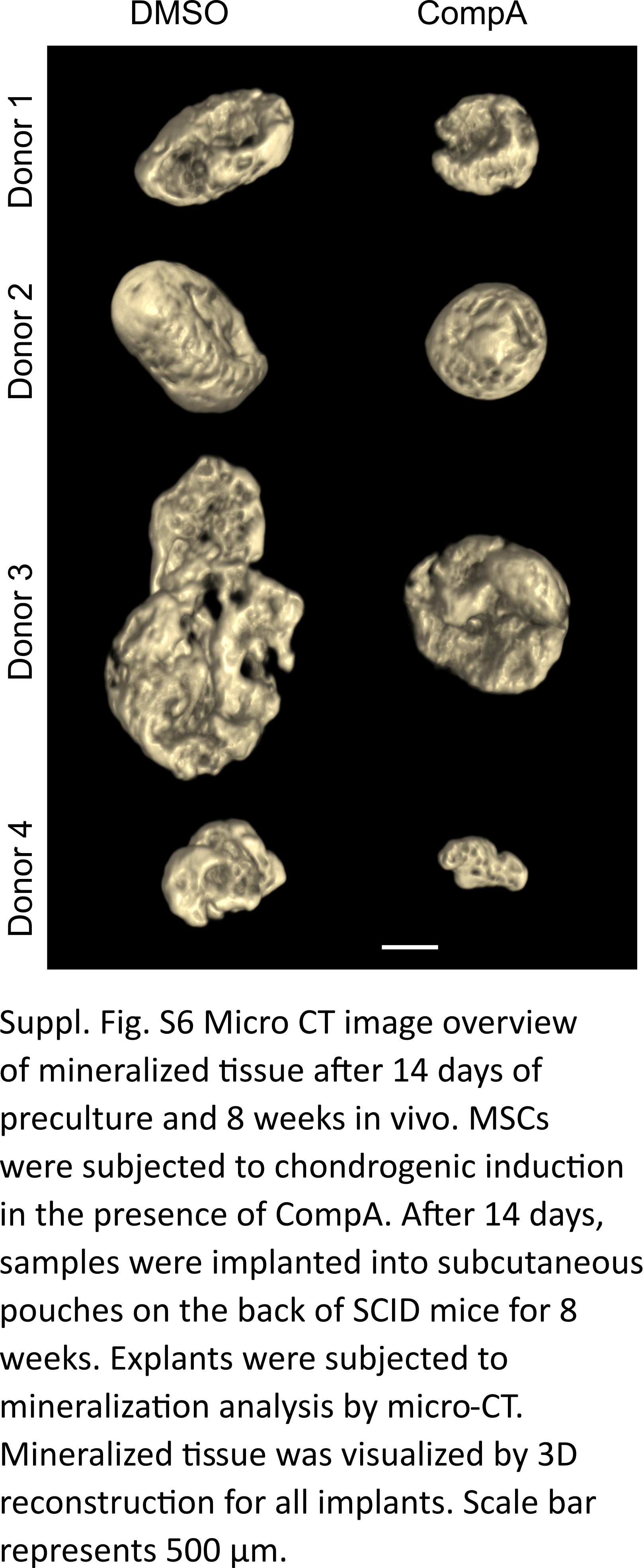

Supplement: Supplementary file 6 — Supplementary Material 6 [file 13287_2024_3710_MOESM6_ESM.png]
