# Supplementary material for: Mesenchymal stromal cell chondrogenesis under ALK1/2/3-specific BMP inhibition: a revision of the prohypertrophic signalling network concept
Source: Stem Cell Res Ther. 2024 Apr 5;15:98. doi: 10.1186/s13287-024-03710-7 (PMC10998299; doi:10.1186/s13287-024-03710-7)
Supplement: Supplementary file 7 — Supplementary Material 7 [file 13287_2024_3710_MOESM7_ESM.pdf]

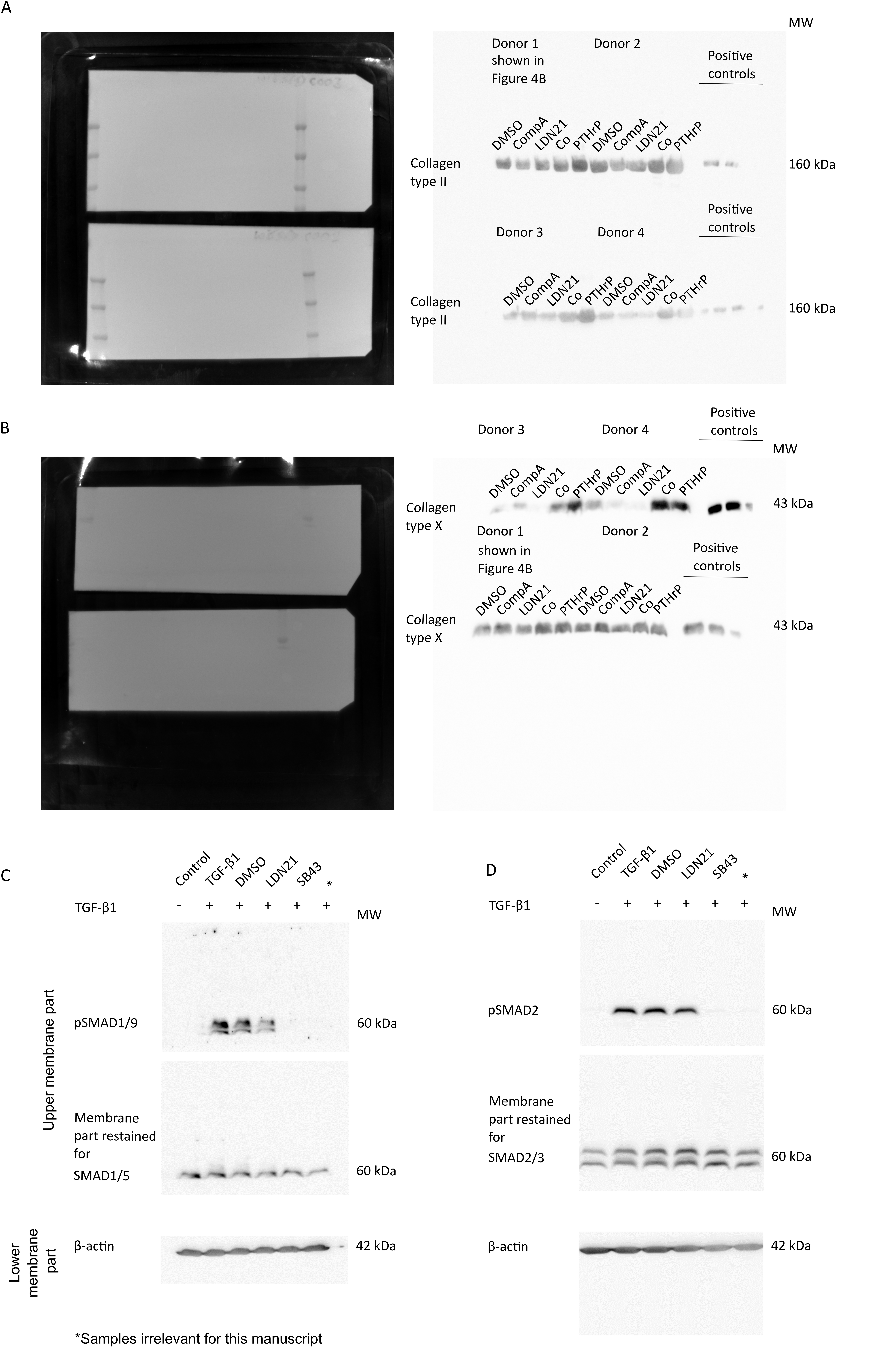


Suppl. Fig. S7 Full-length blots of the cropped Western blot images shown in the main figures. (A, B) Whole membranes of the collagen blots shown in Fig. 4B. (C, D) Whole membranes of the SMAD blots shown in Fig. 7.
